# Supplementary material for: Assessing Phenotypic Variability in Some Eastern European Insular Populations of the Climatic Relict Ilex aquifolium L
Source: Plants (Basel). 2022 Aug 3;11(15):2022. doi: 10.3390/plants11152022 (PMC9370372; doi:10.3390/plants11152022)
Supplement: Supplementary file 1 [file plants-11-02022-s001.zip › Table S5 - Bioclimatic variables and Mantel test.pdf]

Supplementary Materials

Table S5 - Bioclimatic variables in for *Ilex aquifolium* population sampling areas and Mantel statistic tests.

**Bioclim variables Location**	**T.min(°C)**	**T.max(°C)**	**Prec.(mm)**
**RO**	21.83406	36.37375	1.143614
**HU**	22.06715	37.03516	1.000000
**SR**	18.73144	34.70834	5.042770
**BG**	20.68237	37.37291	1.453438

Bioclimatic variables were extracted from https://www.worldclim.org/data/monthlywth.html(2010-2018/tmin_2010-2018/tmax_2010-2018/prec_2010-2018).

Morphological and chemical data - Mantel statistic r: -0.7435; Significance: 1.
GPS and climate data - Mantel statistic r: -0.06895; Significance: 0.33333.
Morphological and GPS data - Mantel statistic r: -0.2876; Significance: 0.58333.
Chemical and GPS data - Mantel statistic r: 0.6264; Significance: 0.25.
Morphological and climate data - Mantel statistic r: 0.7889; Significance: 0.083333.
Chemical and climate data - Mantel statistic r: -0.5417; Significance: 0.91667.
