# Supplementary material for: Assessing Phenotypic Variability in Some Eastern European Insular Populations of the Climatic Relict Ilex aquifolium L
Source: Plants (Basel). 2022 Aug 3;11(15):2022. doi: 10.3390/plants11152022 (PMC9370372; doi:10.3390/plants11152022)
Supplement: Supplementary file 1 [file plants-11-02022-s001.zip › Tables S1-S2-S3-S4 -Phytochemicals identified in the Ilex extracts.pdf]

Supplementary Tables.

Phytochemicals identified in the *Ilex* extracts (Rt –retention time; M[+] – positive ionization specific molecular ion mass; M[-] – negative ionization specific molecular ion mass; (green) specific molecule confirmed by standards.

Table S1. Identified phytochemicals of the Serbian *Ilex* sample

No.	Molecule	Formula	RT	M [+]	M [-]	Fragment 1	Fragment 2	Fragment 3	Fragment 4	Fragment 5	Fragment 6
1	Quinic acid	C7H12O6	1.21		191.05557	173.0443	127.0388	111.0437	109.0281	93.0331	85.0280
2	Glutamic acid	C5H9NO4	1.23	148.06043		130.0500	102.0554	84.0449	56.0502		
3	Adenine (B4)	C5H5N5	1.34	136.06177		119.0357	94.0406	67.0296			
4	Nicotinic acid (Niacin,B3)	C6H5NO2	1.41	124.03930		96.0449	80.0500	78.0344			
5	Pyridoxine (B6)	C8H11NO3	1.47	170.08117		152.0706	134.0601	124.0758	96.0815		
6	Citric acid	C6H8O7	1.54		191.01973	173.0080	129.0181	111.0074	87.0072	85.0280	57.0330
7	Isoleucine or Leucine	C6H13NO2	1.88	132.10191		86.0969	69.0705				
8	Phenylalanine	C9H11NO2	3.20	166.08626		149.0598	131.0492	120.0810	107.0493	103.0546	93.0703
9	Tryptophan	C11H12N2O2	8.07	205.09715		188.0706	170.0600	159.0917	146.0600	132.0809	118.0653
10	Neochlorogenic acid (5-O-Caffeoylquinic acid)	C16H18O9	8.89	355.10236		163.0389	145.0287	135.0441	117.0335	107.0495	89.0389
11	cis-5-O-(4-Coumaroyl)quinic acid	C16H18O8	11.89		337.09289	191.0554	173.0446	163.0389	119.0488	93.0330	
12	trans-5-O-(4-Coumaroyl)quinic acid	C16H18O8	12.56		337.09289	191.0555	173.0445	163.0390	119.0489	93.0331	
13	Chlorogenic acid (3-O-Caffeoylquinic acid)	C16H18O9	14.28	355.10236		163.0390	145.0285	135.0442	117.0338	107.0498	89.0390
14	3-O-Feruloylquinic acid	C17H20O9	14.57		367.10346	193.0500	191.0557	173.0444	134.0361	93.0331	
15	trans-Melilotoside (trans-Glucosyl-2-hydroxycinnamate)	C15H18O8	14.69		325.09289	163.0389	119.0488				
16	Dihydroxycoumarin-O-glucoside	C15H16O9	14.90	341.08671		179.0339	151.0387	133.0286	123.0439	85.0286	

17	Chryptochlorogenic acid (4-O-Caffeoylquinic acid)	C16H18O9	15.64	355.10236		163.0389	145.0284	135.0441	117.0338	107.0497	89.0391
18	cis-3-O-(4-Coumaroyl)quinic acid	C16H18O8	15.67		337.09289	191.0555	173.0445	163.0390	119.0488	93.0330	
19	cis-4-O-(4-Coumaroyl)quinic acid	C16H18O8	16.88		337.09289	191.0554	173.0445	163.0390	119.0488	93.0331	
20	trans-3-O-(4-Coumaroyl)quinic acid	C16H18O8	17.57		337.09289	191.0555	173.0445	163.0389	119.0488	93.0330	
21	1-Benzofuranecarbaldehyde	C9H6O2	17.58	147.04406		119.0493	91.0547	65.0394			
22	Quercetin-3-O-rutinoside-7-O-glucoside	C33H40O21	17.62		771.19893	609.1446	463.0933	462.0807	301.0352	300.0281	299.0198
23	12-Hydroxyjasmonic acid-12-O-glucoside or Tuberonic acid glucoside	C18H28O9	17.64		387.16606	207.1025	163.1118	119.0335	101.0231	89.0228	59.0123
24	5-O-Feruloylquinic acid	C17H20O9	18.00		367.10346	193.0503	191.0555	173.0444	134.0362	93.0331	
25	4-O-Feruloylquinic acid	C17H20O9	18.54		367.10346	193.0499	191.0554	173.0445	134.0362	93.0330	
26	Riboflavin (B2)	C17H20N4O6	18.66	377.14556		359.1339	243.0874	200.0814	172.0869	99.0443	69.0341
27	Indole-4-carbaldehyde	C9H7NO	19.06	146.06004		118.0653	117.0577	91.0547			
28	trans-4-O-(4-Coumaroyl)quinic acid	C16H18O8	19.19		337.09289	191.0554	173.0446	163.0389	119.0489	93.0331	
29	Quercetin-O-(rhamnosyl)hexoside-O-hexoside isomer	C33H40O21	20.60		771.19893	609.1447	463.0888	301.0361	300.0275	271.0251	151.0022
30	Di-O-caffeoylquinic acid isomer 1	C25H24O12	22.37		515.11950	353.0877	191.0553	179.0339	173.0443	135.0439	
31	Eriodictyol-O-hexoside	C21H22O11	22.50		449.10893	287.0563	151.0024	135.0440	107.0124		
32	Isoquercitrin (Hirsutrin, Quercetin-3-O-glucoside)	C21H20O12	22.97		463.08820	301.0362	300.0278	271.0251	255.0301	178.9979	151.0024
33	Rutin	C27H30O16	23.04	611.16066		465.1025	303.0497	145.0493	129.0548	85.0289	71.0497
34	Reinutrin (Reynoutrin, Quercetin-3-O-xyloside)	C20H18O11	23.28		433.07763	301.0361	300.0280	271.0255	255.0297	151.0024	
35	Di-O-caffeoylquinic acid isomer 2	C25H24O12	24.22		515.11950	353.0878	191.0560	179.0345	173.0445	135.0438	
36	Kaempferol-O-hexoside	C21H20O11	24.74		447.09328	285.0398	284.0332	255.0296	227.0338		

37	Kaempferol-O-(rhamnosyl)hexoside	C27H30O15	24.89		593.15119	285.0407	284.0328	255.0299	229.0503	227.0345	
38	Isorhamnetin-3-O-glucoside	C22H22O12	24.97		477.10330	315.0507	314.0437	299.0198	271.0253	243.0294	
39	Isorhamnetin-3-O-rutinoside (Narcissin)	C28H32O16	25.25		623.16176	315.0514	314.0436	300.0278	299.0200	271.0251	243.0296
40	Abscisic acid	C15H20O4	25.40		263.12888	219.1380	204.1150	152.0833	151.0753	139.0748	
41	Naringenin	C15H12O5	27.26		271.06065	177.0169	165.0179	151.0024	119.0489	107.0125	93.0330
42	Homoeriodictyol (3'-Methoxy-4',5,7-trihydroxyflavanone)	C16H14O6	27.33		301.07176	151.0025	149.0596	134.0362	83.0124		
43	Mateglycoside B (Ilexpernoside H, Matenoside A) or Mateglycoside B'	C47H76O18	33.44		927.49535	909.4766	781.4386	765.4436	619.3856	601.3753	487.3444
44	Mateglycoside B (Ilexpernoside H, Matenoside A) or Mateglycoside B'	C47H76O18	34.20		927.49535	909.4878	781.4379	765.4434	619.3852	601.3756	487.3445
45	Matesaponin 3 (Araliasaponin X) or Matesaponin 3'	C53H86O22	35.14		1073.55325	911.5004	749.4501	603.3917	471.3482		
46	Matesaponin 3 (Araliasaponin X) or Matesaponin 3'	C53H86O22	35.55		1073.55325	911.5011	749.4450	603.3912	471.3489		
47	Matesaponin 1 or Matesaponin 1'	C47H76O17	35.84		911.50043	765.4424	749.4487	731.4382	603.3910	585.3803	471.3482
48	Matesaponin 4 or Mateglycoside A (Matenoside C)	C59H96O26	36.10		1219.61116	895.5074	749.4395	733.4545	587.3950	569.3853	455.3526
49	Mateglycoside C or Mateglycoside C'	C41H66O13	36.27		765.44252	747.4325	603.3907	585.3803	471.3469	453.3361	
50	Matesaponin 1 or Matesaponin 1'	C47H76O17	36.56		911.50043	765.4434	749.4487	731.4392	603.3912	585.3804	471.3483
51	Mateglycoside C or Mateglycoside C'	C41H66O13	37.00		765.44252	747.4404	603.3911	585.3801	471.3474	453.3422	

52	Matesaponin 2 or Matesaponin 2'	C53H86O21	37.37		1057.55834	895.5086	733.4539	455.3523			
53	Matesaponin 2 or Matesaponin 2'	C53H86O21	37.97		1057.55834	895.5063	733.4566	587.3939	455.3536		
54	12-Oxo phytodienoic acid	C18H28O3	39.44		291.19657	273.1870	247.2065	165.1273			
55	Hexadecanedioic acid	C16H30O4	40.39		285.20713	267.1969	223.2060				
56	Mateglycoside D (J3a) or J3b	C47H76O16	41.06		895.50552	733.4465	587.3963	569.3862	455.3532		
57	Mateglycoside D (J3a) or J3b	C47H76O16	41.83		895.50552	733.4591	587.3939	569.3840	455.3538		
58	Betulinic acid	C30H48O3	44.51		455.35307	208.1608					
59	Oleanolic acid	C30H48O3	44.95		455.35307	407.3327					
60	Ursolic acid	C30H48O3	45.08		455.35307	407.3324					
61	Uvaol	C30H50O2	45.13	443.38836		425.3765	413.3770	395.3664	235.2056	217.0951	191.1794

Table S2. Identified phytochemicals of the Hungarian *Ilex* sample

No.	Molecule	Formula	RT	M [+]	M [-]	Fragment 1	Fragment 2	Fragment 3	Fragment 4	Fragment 5	Fragment 6
1	Quinic acid	C7H12O6	1.20		191.05557	173.0443	127.0387	111.0438	109.0282	93.0331	85.0280
2	Glutamic acid	C5H9NO4	1.24	148.06043		130.0500	102.0553	84.0449	56.0502		
3	Adenine (B4)	C5H5N5	1.36	136.06177		119.0354	94.0406	67.0296			
4	Nicotinic acid (Niacin,B3)	C6H5NO2	1.45	124.03930		96.0448	80.0501	78.0344			
5	Pyridoxine (B6)	C8H11NO3	1.47	170.08117		152.0706	134.0602	124.0759	96.0810		
6	Citric acid	C6H8O7	1.55		191.01973	173.0077	129.0181	111.0074	87.0072	85.0280	57.0330
7	Isoleucine or Leucine	C6H13NO2	1.88	132.10191		86.0969	69.0705				
8	Phenylalanine	C9H11NO2	3.22	166.08626		149.0597	131.0494	120.0810	107.0494	103.0546	93.0701
9	Tryptophan	C11H12N2O2	8.04	205.09715		188.0707	170.0600	159.0916	146.0600	132.0810	118.0653
10	Neochlorogenic acid (5-O-Caffeoylquinic acid)	C16H18O9	8.83	355.10236		163.0389	145.0287	135.0441	117.0335	107.0495	89.0389

11	cis-5-O-(4-Coumaroyl)quinic acid	C16H18O8	11.87		337.09289	191.0555	173.0447	163.0390	119.0489	93.0330	
12	trans-5-O-(4-Coumaroyl)quinic acid	C16H18O8	12.54		337.09289	191.0555	173.0446	163.0390	119.0489	93.0330	
13	Chlorogenic acid (3-O-Caffeoylquinic acid)	C16H18O9	14.27	355.10236		163.0389	145.0284	135.0442	117.0337	107.0494	89.0390
14	3-O-Feruloylquinic acid	C17H20O9	14.54		367.10346	193.0500	191.0558	173.0445	134.0361	93.0332	
15	trans-Melilotoside (trans-Glucosyl-2-hydroxycinnamate)	C15H18O8	14.68		325.09289	163.0390	119.0488				
16	Dihydroxycoumarin-O-glucoside	C15H16O9	14.90	341.08671		179.0338	151.0392	133.0287	123.0442	85.0285	
17	Chryptochlorogenic acid (4-O-Caffeoylquinic acid)	C16H18O9	15.63	355.10236		163.0389	145.0285	135.0441	117.0337	107.0495	89.0392
18	cis-3-O-(4-Coumaroyl)quinic acid	C16H18O8	15.65		337.09289	191.0556	173.0446	163.0390	119.0489	93.0331	
19	cis-4-O-(4-Coumaroyl)quinic acid	C16H18O8	16.87		337.09289	191.0555	173.0446	163.0390	119.0489	93.0331	
20	trans-3-O-(4-Coumaroyl)quinic acid	C16H18O8	17.55		337.09289	191.0556	173.0445	163.0390	119.0489	93.0331	
21	1-Benzofuranecarbaldehyde	C9H6O2	17.56	147.04406		119.0493	91.0547	65.0393			
22	Quercetin-3-O-rutinoside-7-O-glucoside	C33H40O21	17.60		771.19893	609.1446	463.0933	462.0807	301.0352	300.0281	299.0198
23	12-Hydroxyjasmonic acid-12-O-glucoside or Tuberonic acid glucoside	C18H28O9	17.64		387.16606	207.1018	163.1118	119.0335	101.0231	89.0228	59.0123
24	5-O-Feruloylquinic acid	C17H20O9	18.00		367.10346	193.0501	191.0556	173.0444	134.0361	93.0331	
25	4-O-Feruloylquinic acid	C17H20O9	18.52		367.10346	193.0500	191.0558	173.0445	134.0361	93.0331	
26	Riboflavin (B2)	C17H20N4O6	18.65	377.14556		359.1344	243.0875	200.0815	172.0866	99.0444	69.0341
27	Feruloylquinic acid isomer 1	C17H20O9	18.98		367.10346	193.0505	191.0556	173.0447	134.0361	93.0331	
28	Indole-4-carbaldehyde	C9H7NO	19.04	146.06004		118.0653	117.0574	91.0547			
29	trans-4-O-(4-Coumaroyl)quinic acid	C16H18O8	19.17		337.09289	191.0555	173.0445	163.0391	119.0489	93.0331	

30	Feruloylquinic acid isomer 2	C17H20O9	19.51		367.10346	193.0501	191.0556	173.0448	134.0359	93.0330	
31	Quercetin-O-(rhamnosyl)hexoside-O-hexoside isomer	C33H40O21	20.60		771.19893	609.1447	463.0888	301.0361	300.0275	271.0251	151.0022
32	Di-O-caffeoylquinic acid isomer 1	C25H24O12	22.37		515.11950	353.0877	191.0553	179.0339	173.0443	135.0439	
33	Isoquercitrin (Hirsutrin, Quercetin-3-O-glucoside)	C21H20O12	22.95		463.08820	301.0361	300.0275	271.0249	255.0299	178.9979	151.0017
34	Rutin	C27H30O16	23.04	611.16066		465.1016	303.0498	145.0495	129.0550	85.0289	71.0497
35	Reinutrin (Reynoutrin, Quercetin-3-O-xyloside)	C20H18O11	23.27		433.07763	301.0348	300.0273	271.0248	255.0297	151.0024	
36	Di-O-caffeoylquinic acid isomer 2	C25H24O12	24.20		515.11950	353.0878	191.0560	179.0345	173.0445	135.0438	
37	Kaempferol-O-(rhamnosyl)hexoside	C27H30O15	24.86		593.15119	285.0407	284.0329	255.0300	229.0500	227.0347	
38	Isorhamnetin-3-O-glucoside	C22H22O12	24.95		477.10330	315.0504	314.0447	299.0212	271.0249	243.0296	
39	Isorhamnetin-3-O-rutinoside (Narcissin)	C28H32O16	25.23		623.16176	315.0515	314.0439	300.0279	299.0201	271.0253	243.0297
40	Abscisic acid	C15H20O4	25.41		263.12888	219.1389	204.1155	152.0833	151.0753	139.0748	
41	Naringenin	C15H12O5	27.24		271.06065	177.0185	165.0179	151.0024	119.0489	107.0123	93.0330
42	Homoeriodictyol (3'-Methoxy-4',5,7-trihydroxyflavanone)	C16H14O6	27.31		301.07176	151.0024	149.0595	134.0364	83.0124		
43	Mateglycoside B (Ilexpernoside H, Matenoside A) or Mateglycoside B'	C47H76O18	33.44		927.49535	909.4766	781.4386	765.4436	619.3856	601.3753	487.3444
44	Mateglycoside B (Ilexpernoside H, Matenoside A) or Mateglycoside B'	C47H76O18	34.20		927.49535	909.4878	781.4379	765.4434	619.3852	601.3756	487.3445
45	Matesaponin 3 (Araliasaponin X) or Matesaponin 3'	C53H86O22	35.16		1073.55325	911.5002	749.4432	603.3918	471.3487		

46	Matesaponin 3 (Araliasaponin X) or Matesaponin 3'	C53H86O22	35.54		1073.55325	911.5077	749.4491	603.3906	471.3465		
47	Matesaponin 1 or Matesaponin 1'	C47H76O17	35.85		911.500425	765.4443	749.4490	731.4407	603.3898	585.3804	471.3481
48	Matesaponin 4 or Mateglycoside A (Matenoside C)	C59H96O26	36.07		1219.61116	895.5070	749.4484	733.4542	587.3963	569.3849	455.3532
49	Mateglycoside C or Mateglycoside C'	C41H66O13	36.25		765.44252	747.4350	603.3907	585.3813	471.3487	453.3347	
50	Matesaponin 1 or Matesaponin 1'	C47H76O17	36.54		911.500425	765.4432	749.4488	731.4379	603.3915	585.3807	471.3483
51	Mateglycoside C or Mateglycoside C'	C41H66O13	36.90		765.44252	747.4404	603.3909	585.3783	471.3480	453.3422	
52	Matesaponin 2 or Matesaponin 2'	C53H86O21	37.35		1057.55834	895.5057	733.4539	455.3532			
53	Matesaponin 2 or Matesaponin 2'	C53H86O21	37.98		1057.55834	895.5071	733.4542	587.3951	455.3536		
54	12-Oxo phytodienoic acid	C18H28O3	39.43		291.19657	273.1862	247.2065	165.1273			
55	Hexadecanedioic acid	C16H30O4	40.37		285.20713	267.1970	223.2062				
56	Mateglycoside D (J3a) or J3b	C47H76O16	41.06		895.50552	733.4517	587.3956	569.3876	455.3537		
57	Mateglycoside D (J3a) or J3b	C47H76O16	41.82		895.50552	733.4518	587.3966	569.3825	455.3531		
58	Betulinic acid	C30H48O3	44.50		455.35307	208.1608					
59	Oleanolic acid	C30H48O3	44.96		455.35307	407.3327					
60	Ursolic acid	C30H48O3	45.06		455.35307	407.3324					
61	Uvaol	C30H50O2	45.12	443.38836		425.3765	413.3766	395.3666	235.2055	217.0948	191.1794

Table S3. Identified phytochemicals of the Bulgarian *Ilex* sample

No.	Molecule	Formula	RT	M [+]	M [-]	Fragment 1	Fragment 2	Fragment 3	Fragment 4	Fragment 5	Fragment 6
1	Quinic acid	C7H12O6	1.21		191.05557	173.0447	127.0388	111.0437	109.0284	93.0331	85.0280

2	Glutamic acid	$C_5H_9NO_4$	1.22	148.06043		130.0500	102.0554	84.0449	56.0502		
3	Adenine (B4)	$C_5H_5N_5$	1.33	136.06177		119.0355	94.0402	67.0296			
4	Nicotinic acid (Niacin,B3)	$C_6H_5NO_2$	1.44	124.03930		96.0449	80.0501	78.0344			
5	Citric acid	$C_6H_8O_7$	1.56		191.01973	173.0084	129.0181	111.0073	87.0072	85.0279	57.0330
6	Isoleucine or Leucine	$C_6H_{13}NO_2$	1.87	132.10191		86.0969	69.0705				
7	Phenylalanine	$C_9H_{11}NO_2$	3.17	166.08626		149.0600	131.0493	120.0810	107.0494	103.0546	93.0705
8	Tryptophan	$C_{11}H_{12}N_2O_2$	8.01	205.09715		188.0707	170.0598	159.0916	146.0600	132.0808	118.0654
9	Neochlorogenic acid (5-O-Caffeoylquinic acid)	$C_{16}H_{18}O_9$	8.88	355.10236		163.0389	145.0287	135.0441	117.0335	107.0495	89.0389
10	cis-5-O-(4-Coumaroyl)quinic acid	$C_{16}H_{18}O_8$	11.90		337.09289	191.0555	173.0446	163.0390	119.0489	93.0330	
11	trans-5-O-(4-Coumaroyl)quinic acid	$C_{16}H_{18}O_8$	12.56		337.09289	191.0555	173.0446	163.0389	119.0488	93.0330	
12	Chlorogenic acid (3-O-Caffeoylquinic acid)	$C_{16}H_{18}O_9$	14.27	355.10236		163.0389	145.0284	135.0442	117.0337	107.0493	89.0389
13	3-O-Feruloylquinic acid	$C_{17}H_{20}O_9$	14.57		367.10346	193.0501	191.0558	173.0449	134.0362	93.0332	
14	trans-Melilotoside (trans-Glucosyl-2-hydroxycinnamate)	$C_{15}H_{18}O_8$	14.68		325.09289	163.0390	119.0488				
15	Dihydroxycoumarin-O-glucoside	$C_{15}H_{16}O_9$	14.89	341.08671		179.0339	151.0387	133.0286	123.0439	85.0286	
16	Chryptochlorogenic acid (4-O-Caffeoylquinic acid)	$C_{16}H_{18}O_9$	15.63	355.10236		163.0389	145.0282	135.0441	117.0332	107.0494	89.0391
17	cis-3-O-(4-Coumaroyl)quinic acid	$C_{16}H_{18}O_8$	15.66		337.09289	191.0554	173.0445	163.0390	119.0489	93.0330	
18	cis-4-O-(4-Coumaroyl)quinic acid	$C_{16}H_{18}O_8$	16.88		337.09289	191.0554	173.0446	163.0390	119.0488	93.0331	
19	1-Benzofuranecarbaldehyde	$C_9H_6O_2$	17.56	147.04406		119.0493	91.0547	65.0393			
20	trans-3-O-(4-Coumaroyl)quinic acid	$C_{16}H_{18}O_8$	17.56		337.09289	191.0558	173.0445	163.0390	119.0489	93.0330	
21	12-Hydroxyjasmonic acid-12-O-glucoside or Tuberonic acid glucoside	$C_{18}H_{28}O_9$	17.63		387.16606	207.1023	163.1118	119.0336	101.0231	89.0229	59.0123
22	5-O-Feruloylquinic acid	$C_{17}H_{20}O_9$	18.01		367.10346	193.0500	191.0555	173.0447	134.0362	93.0331	

23	4-O-Feruloylquinic acid	C17H20O9	18.53		367.10346	193.0502	191.0548	173.0445	134.0360	93.0330	
24	Riboflavin (B2)	C17H20N4O6	18.64	377.14556		359.1353	243.0874	200.0824	172.0869	99.0444	69.0341
25	Feruloylquinic acid isomer	C17H20O9	18.97		367.10346	193.0501	191.0547	173.0444	134.0359	93.0330	
26	Indole-4-carbaldehyde	C9H7NO	19.03	146.06004		118.0654	117.0573	91.0547			
27	trans-4-O-(4-Coumaroyl)quinic acid	C16H18O8	19.17		337.09289	191.0554	173.0445	163.0389	119.0487	93.0330	
28	Di-O-caffeoylquinic acid isomer 1	C25H24O12	22.36		515.11950	353.0877	191.0553	179.0339	173.0443	135.0439	
29	Rutin	C27H30O16	23.05	611.16066		465.1025	303.0496	145.0496	129.0547	85.0289	71.0498
30	Di-O-caffeoylquinic acid isomer 2	C25H24O12	24.22		515.11950	353.0878	191.0560	179.0345	173.0445	135.0438	
31	Kaempferol-O-(rhamnosyl)hexoside	C27H30O15	24.88		593.15119	285.0409	284.0333	255.0300	229.0501	227.0352	
32	Isorhamnetin-3-O-glucoside	C22H22O12	25.07		477.10330	315.0507	314.0437	299.0198	271.0253	243.0294	
33	Isorhamnetin-3-O-rutinoside (Narcissin)	C28H32O16	25.24		623.16176	315.0514	314.0436	300.0278	299.0200	271.0251	243.0296
34	Abscisic acid	C15H20O4	25.42		263.12888	219.1377	204.1154	152.0831	151.0752	139.0748	
35	Naringenin	C15H12O5	27.24		271.06065	177.0169	165.0179	151.0024	119.0489	107.0125	93.0330
36	Homoeriodictyol (3'-Methoxy-4',5,7-trihydroxyflavanone)	C16H14O6	27.33		301.07176	151.0025	149.0596	134.0362	83.0124		
37	Mateglycoside B (Ilexpernoside H, Matenoside A) or Mateglycoside B'	C47H76O18	34.21		927.49535	909.4878	781.4394	765.4437	619.3857	601.3746	487.3445
38	Matesaponin 3 (Araliasaponin X) or Matesaponin 3'	C53H86O22	35.15		1073.55325	911.5004	749.4522	603.3917	471.3514		
39	Matesaponin 3 (Araliasaponin X) or Matesaponin 3'	C53H86O22	35.55		1073.55325	911.5011	749.4450	603.3912	471.3489		
40	Matesaponin 4 or Mateglycoside A (Matenoside C)	C59H96O26	36.09		1219.61116	895.5073	749.4509	733.4553	587.3942	569.3846	455.3526

41	Mateglycoside C or Mateglycoside C'	C41H66O13	36.27		765.44252	747.4325	603.3907	585.3803	471.3469	453.3361	
42	Matesaponin 1 or Matesaponin 1'	C47H76O17	36.58		911.500425	765.4429	749.4487	731.4385	603.3911	585.3794	471.3482
43	Mateglycoside C or Mateglycoside C'	C41H66O13	36.92		765.44252	747.4319	603.3918	585.3801	471.3488	453.3422	
44	Matesaponin 2 or Matesaponin 2'	C53H86O21	37.36		1057.55834	895.5086	733.4543	455.3575			
45	Matesaponin 2 or Matesaponin 2'	C53H86O21	37.97		1057.55834	895.5063	733.4566	587.3939	455.3536		
46	Mateglycoside D (J3a) or J3b	C47H76O16	40.98		895.50552	733.4551	587.3948	569.3862	455.3505		
47	Mateglycoside D (J3a) or J3b	C47H76O16	41.81		895.50552	733.4543	587.3973	569.3867	455.3536		
48	Betulinic acid	C30H48O3	44.51		455.35307	208.1608					
49	Oleanolic acid	C30H48O3	44.95		455.35307	407.3327					
50	Ursolic acid	C30H48O3	45.08		455.35307	407.3324					

Table S4. Identified phytochemicals of the Romanian *Ilex* sample

No.	Molecule	Formula	RT	M [+]	M [-]	Fragment 1	Fragment 2	Fragment 3	Fragment 4	Fragment 5	Fragment 6
1	Quinic acid	C7H12O6	1.20		191.05557	173.0444	127.0388	111.0436	109.0282	93.0331	85.0280
2	Glutamic acid	C5H9NO4	1.24	148.06043		130.0500	102.0553	84.0448	56.0502		
3	Adenine (B4)	C5H5N5	1.33	136.06177		119.0354	94.0404	67.0290			
4	Nicotinic acid (Niacin,B3)	C6H5NO2	1.34	124.03930		96.0449	80.0500	78.0345			
5	Pyridoxine (B6)	C8H11NO3	1.46	170.08117		152.0705	134.0601	124.0757	96.0811		
6	Citric acid	C6H8O7	1.52		191.01973	173.0080	129.0181	111.0073	87.0072	85.0279	57.0330
7	Isoleucine or Leucine	C6H13NO2	1.86	132.10191		86.0969	69.0705				
8	Phenylalanine	C9H11NO2	3.19	166.08626		149.0598	131.0492	120.0809	107.0494	103.0547	93.0701
9	Tryptophan	C11H12N2O2	8.06	205.09715		188.0707	170.0600	159.0916	146.0600	132.0810	118.0653

10	Neochlorogenic acid (5-O-Caffeoylquinic acid)	C16H18O9	8.81	355.10236		163.0389	145.0284	135.0441	117.0337	107.0494	89.0389
11	cis-5-O-(4-Coumaroyl)quinic acid	C16H18O8	11.87		337.09289	191.0555	173.0445	163.0389	119.0488	93.0331	
12	trans-5-O-(4-Coumaroyl)quinic acid	C16H18O8	12.56		337.09289	191.0556	173.0445	163.0390	119.0489	93.0330	
13	Chlorogenic acid (3-O-Caffeoylquinic acid)	C16H18O9	14.27	355.10236		163.0389	145.0284	135.0442	117.0337	107.0495	89.0390
14	3-O-Feruloylquinic acid	C17H20O9	14.57		367.10346	193.0501	191.0555	173.0446	134.0362	93.0331	
15	trans-Melilotoside (trans-Glucosyl-2-hydroxycinnamate)	C15H18O8	14.69		325.09289	163.0390	119.0489				
16	Dihydroxycoumarin-O-glucoside	C15H16O9	14.89	341.08671		179.0339	151.0395	133.0284	123.0440	85.0289	
17	Chryptochlorogenic acid (4-O-Caffeoylquinic acid)	C16H18O9	15.61	355.10236		163.0389	145.0284	135.0442	117.0337	107.0494	89.0390
18	cis-3-O-(4-Coumaroyl)quinic acid	C16H18O8	15.66		337.09289	191.0555	173.0445	163.0390	119.0489	93.0330	
19	cis-4-O-(4-Coumaroyl)quinic acid	C16H18O8	16.89		337.09289	191.0553	173.0444	163.0389	119.0488	93.0330	
20	trans-3-O-(4-Coumaroyl)quinic acid	C16H18O8	17.58		337.09289	191.0559	173.0445	163.0390	119.0489	93.0331	
21	1-Benzofuranecarbaldehyde	C9H6O2	17.59	147.04406		119.0493	91.0547	65.0392			
22	12-Hydroxyjasmonic acid-12-O-glucoside or Tuberonic acid glucoside	C18H28O9	17.64		387.16606	207.1021	163.1118	119.0336	101.0233	89.0229	59.0123
23	Quercetin-3-O-rutinoside-7-O-glucoside	C33H40O21	17.64		771.19893	609.1473	463.0933	462.0801	301.0365	300.0270	299.0199
24	5-O-Feruloylquinic acid	C17H20O9	18.01		367.10346	193.0502	191.0555	173.0446	134.0362	93.0331	
25	4-O-Feruloylquinic acid	C17H20O9	18.55		367.10346	193.0500	191.0558	173.0445	134.0362	93.0330	
26	Riboflavin (B2)	C17H20N4O6	18.64	377.14556		359.1343	243.0875	200.0823	172.0868	99.0443	69.0341
27	Indole-4-carbaldehyde	C9H7NO	19.03	146.06004		118.0653	117.0577	91.0547			
28	trans-4-O-(4-Coumaroyl)quinic acid	C16H18O8	19.20		337.09289	191.0554	173.0446	163.0390	119.0489	93.0331	

29	Quercetin-O-(rhamnosyl)hexoside-O-hexoside isomer	C33H40O21	20.59		771.19893	609.1478	463.0888	301.0356	300.0276	271.0249	151.0026
30	Quercetin-O-(pentosyl)hexoside	C26H28O16	21.23	597.14501		465.1025	303.0497	257.0443	165.0182	153.0180	85.0289
31	Di-O-caffeoylquinic acid isomer 1	C25H24O12	22.36		515.11950	353.0881	191.0555	179.0341	173.0446	135.0440	
32	Eriodictyol-O-hexoside	C21H22O11	22.50		449.10893	287.0563	151.0024	135.0440	107.0124		
33	Isoquercitrin (Hirsutrin, Quercetin-3-O-glucoside)	C21H20O12	22.96		463.08820	301.0360	300.0278	271.0250	255.0298	178.9973	151.0028
34	Rutin	C27H30O16	23.04	611.16066		465.1015	303.0497	145.0495	129.0547	85.0289	71.0497
35	Reinutrin (Reynoutrin, Quercetin-3-O-xyloside)	C20H18O11	23.27		433.07763	301.0355	300.0278	271.0245	255.0301	151.0024	
36	Di-O-caffeoylquinic acid isomer 2	C25H24O12	24.19		515.11950	353.0882	191.0555	179.0341	173.0446	135.0440	
37	Kaempferol-O-hexoside	C21H20O11	24.76		447.09328	285.0398	284.0332	255.0296	227.0338		
38	Kaempferol-O-(rhamnosyl)hexoside	C27H30O15	24.88		593.15119	285.0409	284.0330	255.0299	229.0501	227.0347	
39	Isorhamnetin-3-O-glucoside	C22H22O12	24.96		477.10330	315.0521	314.0438	299.0197	271.0254	243.0293	
40	Isorhamnetin-3-O-rutinoside (Narcissin)	C28H32O16	25.24		623.16176	315.0515	314.0437	300.0278	299.0200	271.0252	243.0296
41	Abscisic acid	C15H20O4	25.41		263.12888	219.1384	204.1151	152.0834	151.0753	139.0757	
42	Naringenin	C15H12O5	27.26		271.06065	177.0181	165.0184	151.0025	119.0488	107.0125	93.0329
43	Homoeriodictyol (3'-Methoxy-4',5,7-trihydroxyflavanone)	C16H14O6	27.33		301.07176	151.0026	149.0596	134.0361	83.0125		
44	Mateglycoside B (Ilexpernoside H, Matenoside A) or Mateglycoside B'	C47H76O18	34.22		927.49535	909.4858	781.4387	765.4437	619.3856	601.3756	487.3444
45	Matesaponin 1 or Matesaponin 1'	C47H76O17	35.93		911.500425	765.4424	749.4502	731.4382	603.3934	585.3841	471.3493
46	Matesaponin 4 or Mateglycoside A (Matenoside C)	C59H96O26	36.09		1219.61116	895.5071	749.4464	733.4540	587.3953	569.3848	455.3534

47	Matesaponin 1 or Matesaponin 1'	C47H76O17	36.56		911.500425	765.4439	749.4489	731.4389	603.3909	585.3803	471.3483
48	Mateglycoside C or Mateglycoside C'	C41H66O13	37.00		765.44252	747.4282	603.3908	585.3796	471.3474	453.3387	
49	Matesaponin 2 or Matesaponin 2'	C53H86O21	37.36		1057.55834	895.5086	733.4543	455.3526			
50	Matesaponin 2 or Matesaponin 2'	C53H86O21	37.97		1057.55834	895.5061	733.4538	587.3991	455.3530		
51	12-Oxo phytodienoic acid	C18H28O3	39.43		291.19657	273.1870	247.2065	165.1273			
52	Hexadecanedioic acid	C16H30O4	40.37		285.20713	267.1967	223.2065				
53	Mateglycoside D (J3a) or J3b	C47H76O16	41.07		895.50552	733.4465	587.3963	569.3862	455.3532		
54	Mateglycoside D (J3a) or J3b	C47H76O16	41.83		895.50552	733.4591	587.3939	569.3840	455.3538		
55	Betulinic acid	C30H48O3	44.51		455.35307	208.1608					
56	Oleanolic acid	C30H48O3	44.96		455.35307	407.3327					
57	Ursolic acid	C30H48O3	45.09		455.35307	407.3324					
58	Uvaol	C30H50O2	45.11	443.38836		425.3765	413.3778	395.3664	235.2056	217.0952	191.1794
