# Supplementary figures and images for: Assessing Phenotypic Variability in Some Eastern European Insular Populations of the Climatic Relict Ilex aquifolium L
Source: Plants (Basel). 2022 Aug 3;11(15):2022. doi: 10.3390/plants11152022 (PMC9370372; doi:10.3390/plants11152022)

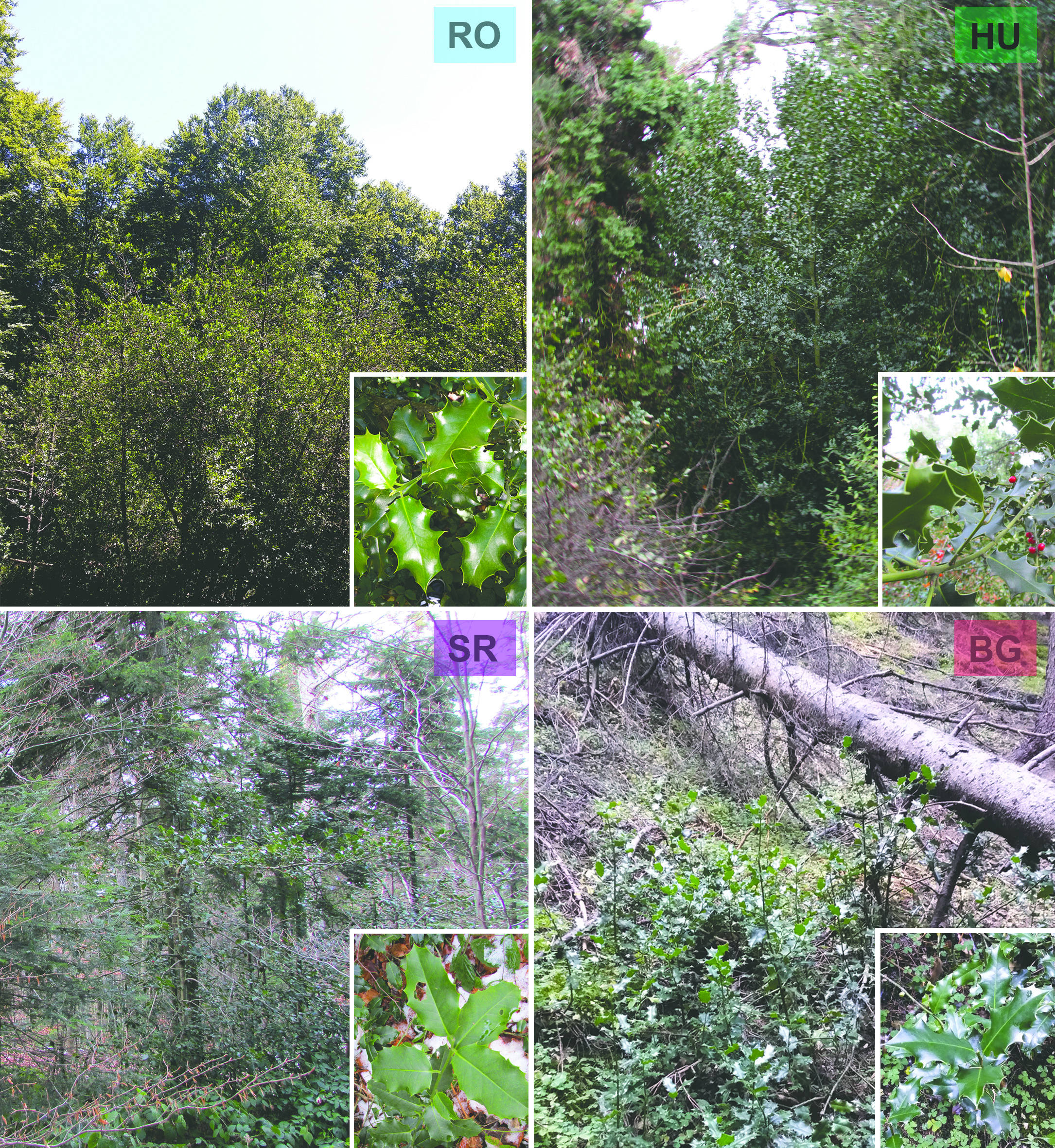

Supplement: Supplementary file 1 [file plants-11-02022-s001.zip › Figure S1 - Ilex aquifolium population habitat and local flora at the RO, HU, SR and BG locations..jpg]

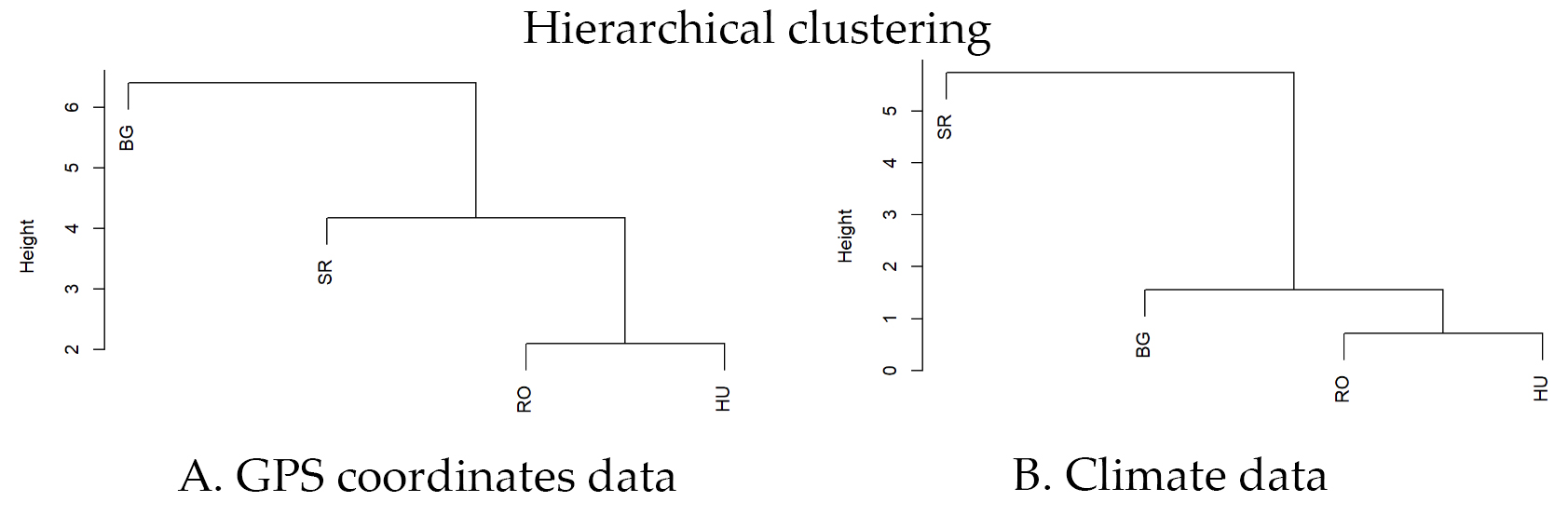

Supplement: Supplementary file 1 [file plants-11-02022-s001.zip › Figure S10 - Dendrograms depicting the hierarchical clustering of GPS coordinates and climate data, respectively..jpg]

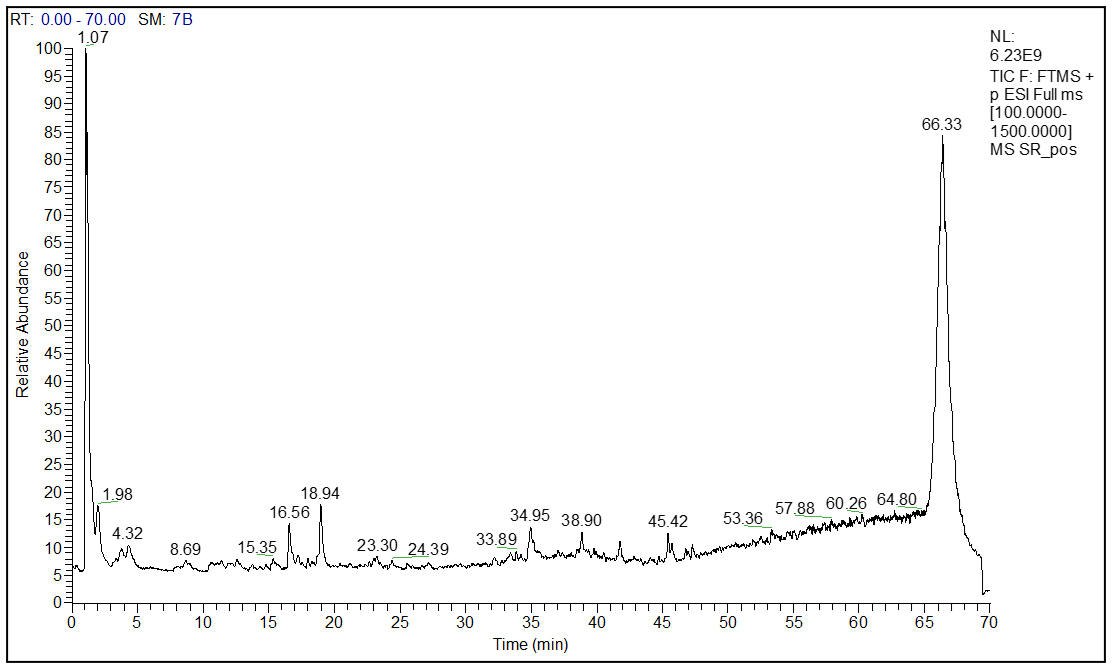

Supplement: Supplementary file 1 [file plants-11-02022-s001.zip › Figure S2 - Total ion chromatogram of Serbian Ilex extract in positive ionization mode..jpg]

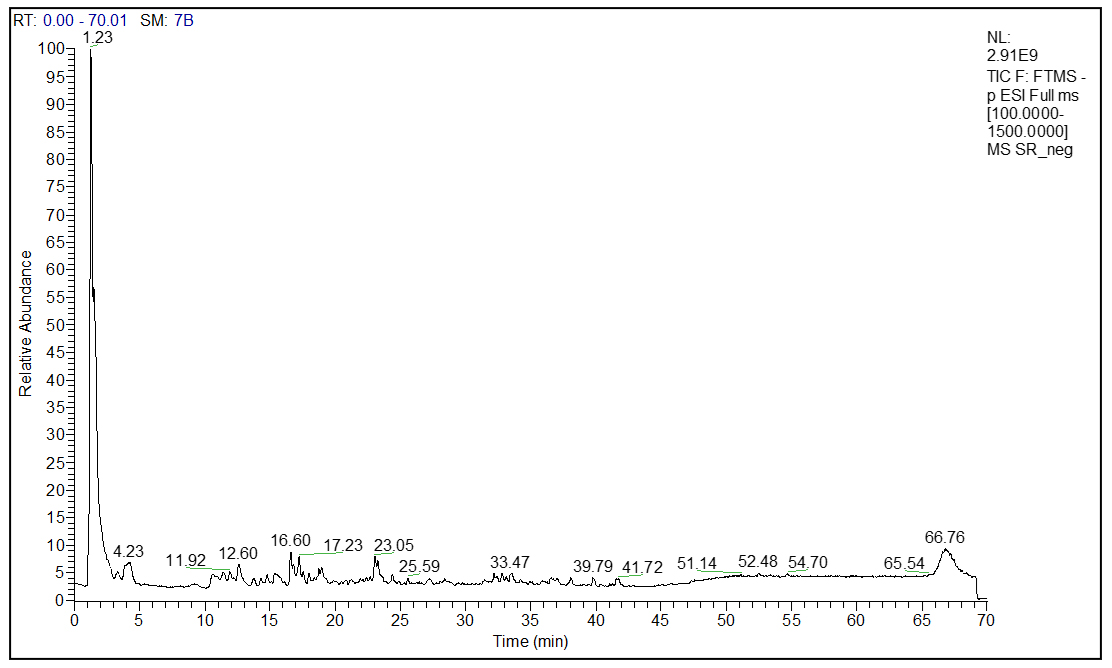

Supplement: Supplementary file 1 [file plants-11-02022-s001.zip › Figure S3 - Total ion chromatogram of Serbian Ilex extract in negative ionization mode..jpg]

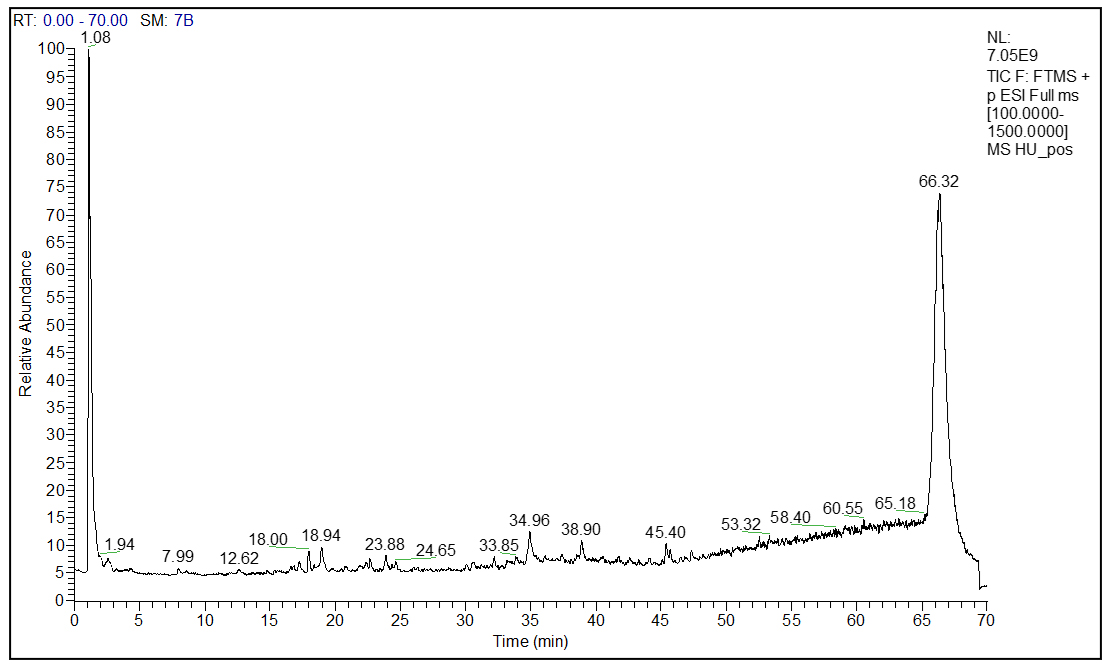

Supplement: Supplementary file 1 [file plants-11-02022-s001.zip › Figure S4 - Total ion chromatogram of Hungarian Ilex extract in positive ionization mode..jpg]

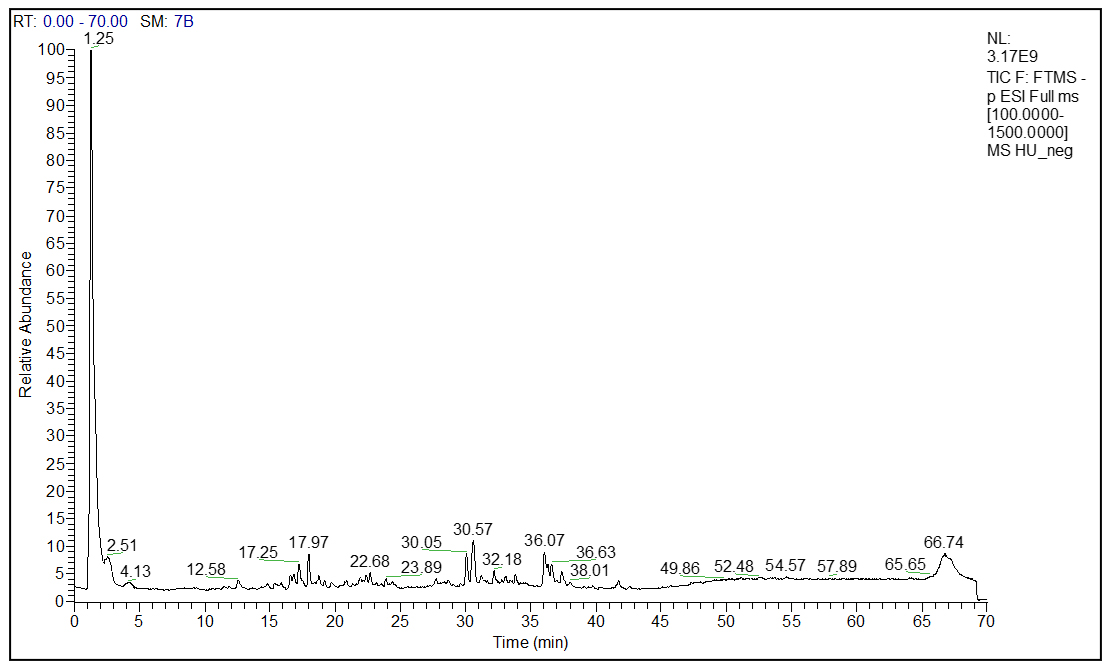

Supplement: Supplementary file 1 [file plants-11-02022-s001.zip › Figure S5 - Total ion chromatogram of Hungarian Ilex extract in negative ionization mode..jpg]

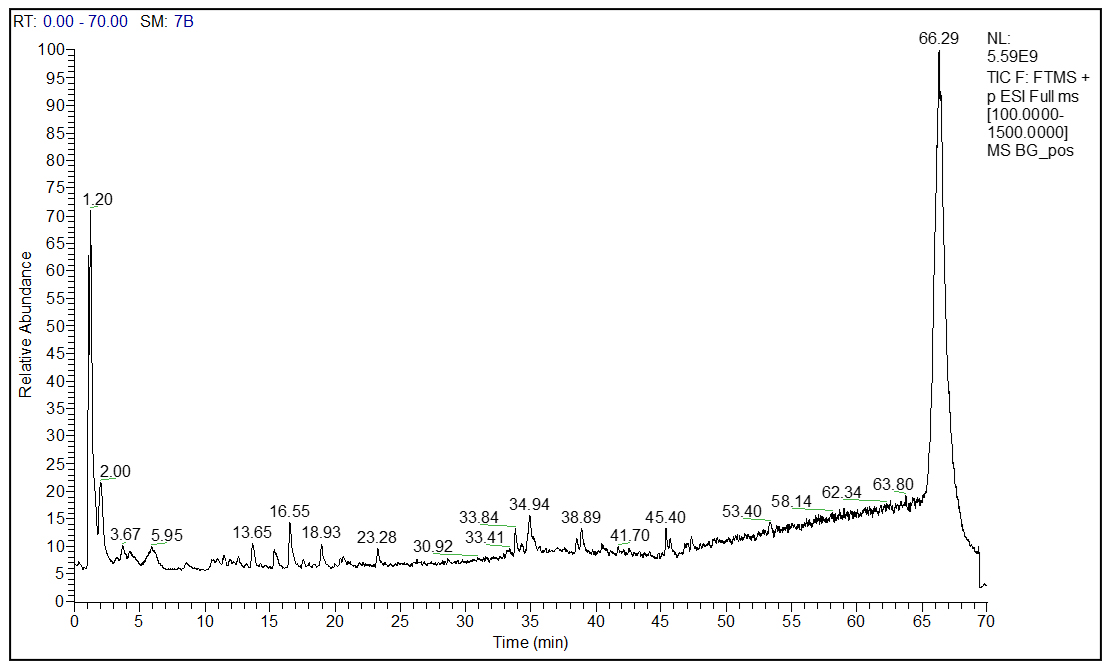

Supplement: Supplementary file 1 [file plants-11-02022-s001.zip › Figure S6 - Total ion chromatogram of Bulgarian Ilex extract in positive ionization mode..jpg]

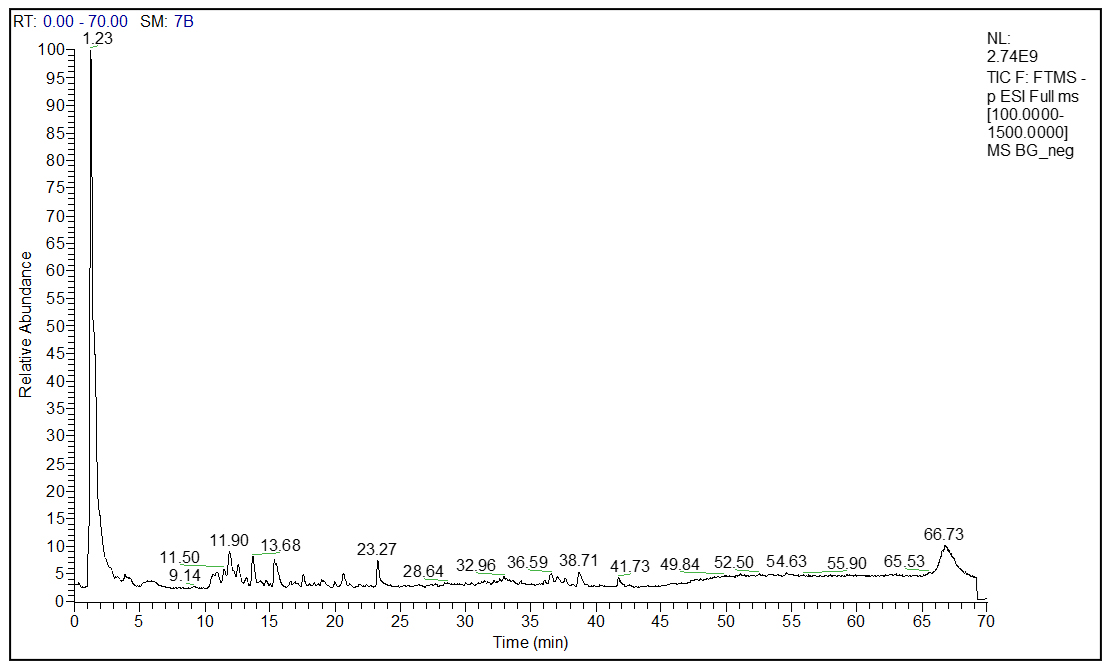

Supplement: Supplementary file 1 [file plants-11-02022-s001.zip › Figure S7 - Total ion chromatogram of Bulgarian Ilex extract in negative ionization mode..jpg]

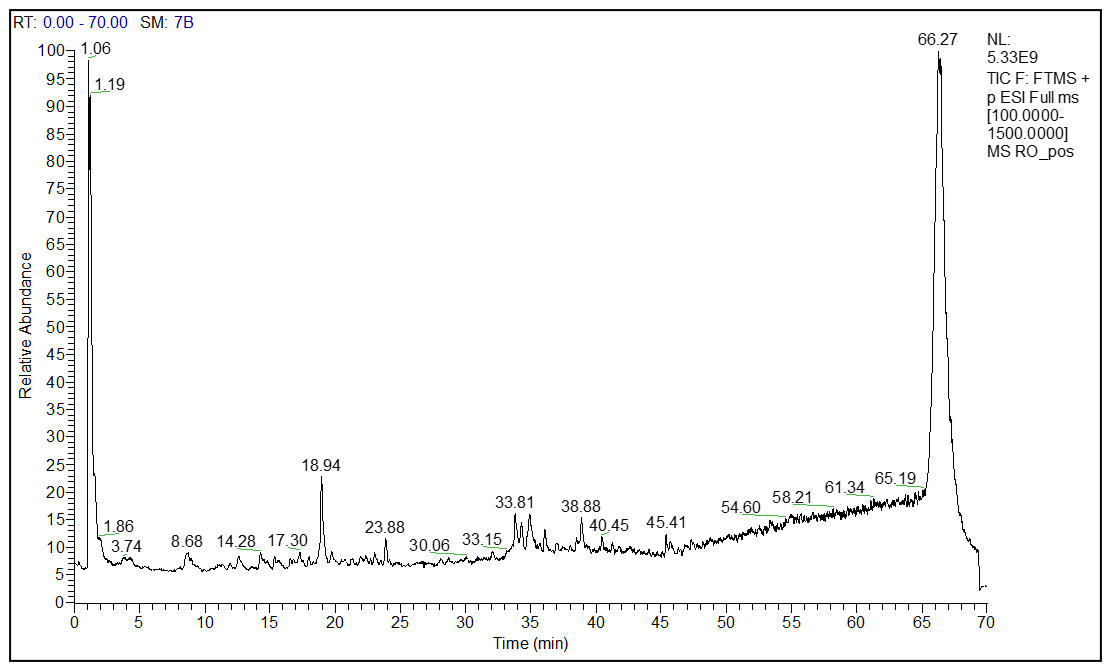

Supplement: Supplementary file 1 [file plants-11-02022-s001.zip › Figure S8 - Total ion chromatogram of Rumanian Ilex extract in positive ionization mode..jpg]

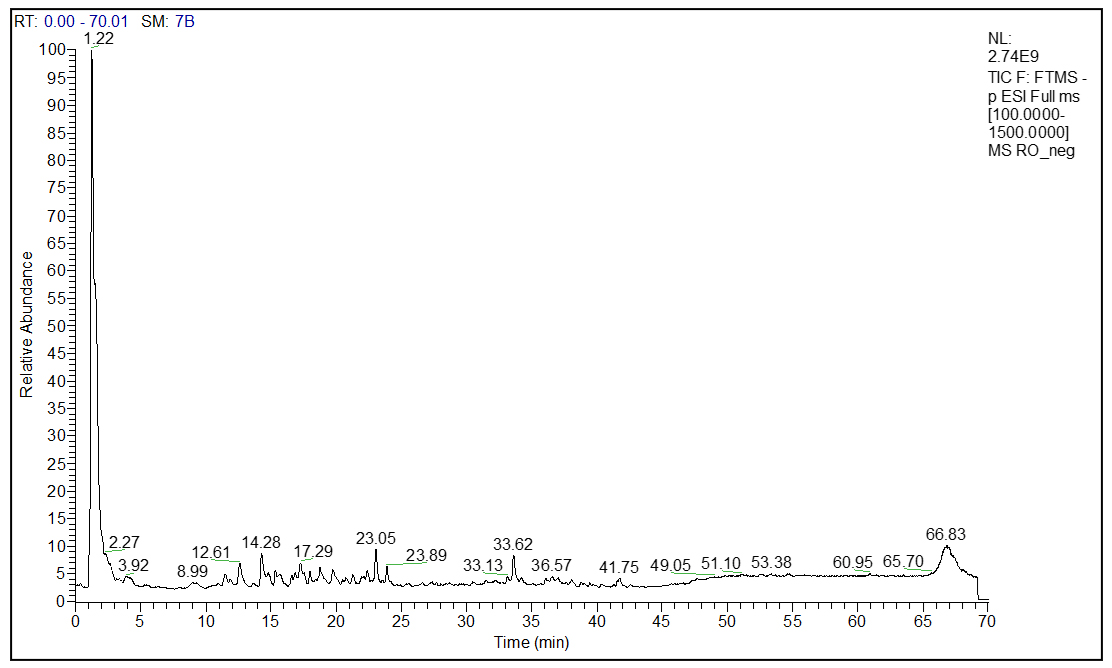

Supplement: Supplementary file 1 [file plants-11-02022-s001.zip › Figure S9 - Total ion chromatogram of Rumanian Ilex extract in negative ionization mode..jpg]
